# Clinical and bacteriological features and prognosis of ascitic fluid infection in Chinese patients with cirrhosis

**DOI:** 10.1186/s12879-018-3101-1

**Published:** 2018-06-04

**Authors:** Nian-zhi Ning, Tao Li, Ju-ling Zhang, Fen Qu, Jie Huang, Xiong Liu, Zhan Li, Wei Geng, Jun-liang Fu, Wang Huan, Shu-yong Zhang, Chun-mei Bao, Hui Wang

**Affiliations:** 1 The State Key Laboratory of Pathogen and Biosecurity, Beijing Institute of Microbiology and Epidemiology, No.20 Dongda Street, Fengtai District, Beijing, 100071 China; 2 Clinical Diagnostic Center, Beijing 302 Hospital, No. 100 Western 4th Middle Ring Road, Beijing, 100039 China; 3 Research Center for Biological Therapy, Institute of Translational Hepatology, Beijing 302 Hospital, Beijing, 100039 China

**Keywords:** Cirrhosis, Spontaneous bacterial peritonitis, Bacterascites, Causative pathogens, Mortality, Risk factor

## Abstract

**Background:**

Spontaneous bacterial peritonitis (SBP) and bacterascites (BA) represent frequent and serious complications in cirrhosis patients with ascites. However, few detailed data are available regarding the clinical and bacteriological feature of SBP or BA patients in China.

**Methods:**

We retrospectively analyzed bacteriological and clinical characteristics of patients with SBP and BA at Beijing 302 Hospital in China from January 2012 to December 2015.

**Results:**

A total of 600 patients with SBP (*n* = 408) or BA (*n* = 192) were enrolled. Patients with BA appeared to have a less severe clinical manifestation and lower mortality rate than patients with SBP. Gram-negative bacteria formed the majority of pathogens in SBP (73.9%) and BA (55.8%) cases. Higher ascitic fluid polymorphonuclear leucocytes (PMN) count and hepatocellular carcinoma were independent risk factors for BA episode progressing to SBP. The concentration of blood urea nitrogen (BUN) was independent risk factor for 30-day mortality of BA patients. For patients with SBP, the independent risk factors for 30-day mortality were age, Model for End-Stage Liver Disease (MELD) score, septic shock and hepatocellular carcinoma. Patients with third-generation cephalosporin or carbapenems resistant infection had a significantly lower survival probability. There were significant differences in clinical characteristics and outcome among the major bacteria. Multivariate analysis showed that patients infected with *Klebsiella* spp. had higher hazard ratio of 30-day mortality.

**Conclusion:**

Our study reported the bacteriological and clinical characteristics of patients with SBP and BA. Higher ascitic fluid PMN count and hepatocellular carcinoma were found to be independent risk factors for BA episode progressed to SBP. Outcome of ascitic fluid infection in patients with cirrhosis was influenced by the type of bacteria and antimicrobial susceptibility.

**Electronic supplementary material:**

The online version of this article (10.1186/s12879-018-3101-1) contains supplementary material, which is available to authorized users.

## Background

Cirrhotic patients with ascites usually face poor outcomes, especially if infections such as spontaneous bacterial peritonitis (SBP) develop [1]. Prompt and appropriate empirical antibiotic therapy must be initiated immediately after the diagnosis of SBP to cover the most commonly isolated bacteria [2]. Previous studies showed that gram-negative bacteria, mainly *Enterobacteriaceae*, were major causative organisms of ascitic fluid infection [3, 4]. Third generation cephalosporins are the first line antibiotics to treat spontaneous bacterial peritonitis; however, it was showed that the initial treatment with cefotaxime, one of the most commonly used cephalosporins, failed more frequently than expected [5]. It may be explained by the change of causative pathogen profile and the emergence of antibiotic-resistant pathogens. In recent years, several studies have reported changes in the epidemiology of causative bacteria in SBP. *Enterococcus* spp. was increasingly recognized as an important pathogen of ascitic fluid infection for patients with cirrhosis. Reuken *et al.* confirmed a profound increase in the frequency of *Enterococcal* infection from 11% to 35% between 2000 and 2011 in a German tertiary center [6]. Piroth *et al.* found that *Enteroccocci* were isolated in 24% of ascitic fluid infection episodes, and in 48% from patients receiving quinolone prophylaxis in four French hospitals [7]. The emergence and spread of multi-drug resistant bacteria such as methicillin-resistant *Staphylococcus aureus* (MRSA), extended-spectrum beta-lactamases (ESBL)-producing *Enterobacteriaceae* and Carbapenemase producing (KPC) *Klebsiella pneumonia* are also of great concern since they may be associated with higher mortality rate [8]. In recent years, an increased prevalence of multi-drug resistant bacteria in SBP cases was reported [9, 10].

Liver diseases affect ~ 300 million people in China, and the incidence of liver cirrhosis has increased during recent years because of the low awareness of the perniciousness of liver diseases and low treatment rate for these patients [11]. Some studies have described the pathogens profile and drug resistance of SBP in cirrhotic patients in China [12, 13]. However, those studies were limited by being single-center study and failed to investigate the characteristics and outcome of SBP. Thus, more timely and comprehensive studies on bacteriological and clinical characteristics of SBP and BA in China are necessary.

In this study, we aimed at assessing the possible changes in bacteria etiology of SBP and BA, the risk factors of 30-day mortality and the differences in clinical characteristics and prognosis among patients with different causative pathogens.

## Methods

### Setting and study design

This study was conducted at Beijing 302 Hospital, which is the largest liver disease hospital in China. The hospital’s database holds records of clinical histories, disease manifestations, physical and laboratory findings, and treatments of admitted patients. Bacteriology laboratory files and patient characteristics were reviewed to identify all cases with positive ascitic fluid cultures in cirrhotic patients hospitalized in our institution from January 1, 2012 to December 31, 2015. Patients with secondary peritonitis were excluded from the study. Patients with a positive culture for common skin contaminants (*coagulase-negative staphylococci*, *corynebacteria*, *propionibacteria*, and *Bacillus* spp*.*) were also excluded.

### Definitions

The diagnosis of cirrhosis was based on clinical, biochemical, histological and/or radiological findings. A diagnostic paracentesis was performed in all patients as recommended by European Association for the Study of the Liver (EASL) [2]. SBP was diagnosed when (a) ascitic fluid polymorphonuclear leucocytes (PMN) count ≥250 cells/μL, (b) ascitic fluid culture was positive; (c) there was no evident intra-abdominal surgically treatable source for infection [2, 14]. The diagnosis of BA was made when (a) the ascitic fluid PMN count < 250 cells/μL, (b) ascitic fluid culture was positive; (c) there was no evident intra-abdominal surgically treatable source for infection [2, 15]. Fever, chills, abdominal pain and abdominal tenderness were considered symptoms of peritonitis [15]. Severity of cirrhosis was assessed at the time of the SBP or BA diagnosis using the Model for End-Stage Liver Disease (MELD) score [16]. Nosocomial infection was defined as an infection that occurred > 48 h after admission to the hospital [17].

### Microbiological methods

Ascitic fluid samples were extracted and inoculated into bottles at the patient’s bedside by aseptic manipulation and cultured with BacT/Alert 240 automated blood culture system (BioMérieux France). White blood cell (WBC) and PMN were counted by Sysmex automatic cell analyzer XT-4000 (SYSMEX, Japan). Microorganism identification was performed using the VITEK-II auto microbe system (BioMérieux France). Isolated pathogens were tested for antimicrobial susceptibility using the disk-diffusion method and minimum inhibitory concentration testing. Antimicrobial susceptibility was judged according to guidelines of the Clinical and Laboratory Standards Institute (CLSI) [18].

### Empirical antibiotic therapy

Third-generation cephalosporins (TGC), such as cefotaxime, were used as empirical therapy antibiotic in our hospital. Empirical antibiotic therapy initiated immediately on all patients with ascitic PMN count ≥250 cells/μL, without the results of ascitic fluid culture. Bacterascites patients exhibit signs of systemic inflammation are also treated with cefotaxime. Otherwise, the bacterascites patient should undergo a second paracentesis when culture results come back positive. Patients in whom the repeat PMN count is ≥250/μL would be treated for SBP. Carbapenems were used in all severe patients with septic shock. Paracentesis was performed repeatedly 2–3 days after initiation of antibiotic therapy to determine leukocyte and PMN counts in ascitic fluid.

### Statistical analysis

Quantitative variables were given as the mean ± standard deviation or the median (interquartile range). Student’s *t* test or Mann–Whitney U-test was used to compare continuous variables, and the x^2^ test or Fisher’s exact test was used to compare categorical variables. Statistical differences among several groups were analyzed using one-way ANOVA or the nonparametric Kruskal-Wallis test, as appropriate. Risk factors for SBP development were determined by multivariate binary logistic regression including significant univariate predictors (*p* < 0.05) using stepwise backward elimination. The life-table method was used to compare survival probability for patients according to different variables. A Cox proportional hazard model analysis was carried out to identify independent predictors of 30-day mortality for every episode, defining death as the main event. Variables with *p* < 0.05 in the univariate analysis were candidates for multivariate analysis and non-significant factors were removed by a backward selection process. All tests were two-tailed and *p* < 0.05 were considered to be statistically significant. IBM SPSS Statistics 19.0 was used for statistical analyses.

## Results

### General characteristics and clinical presentation

**Fig. 1:**
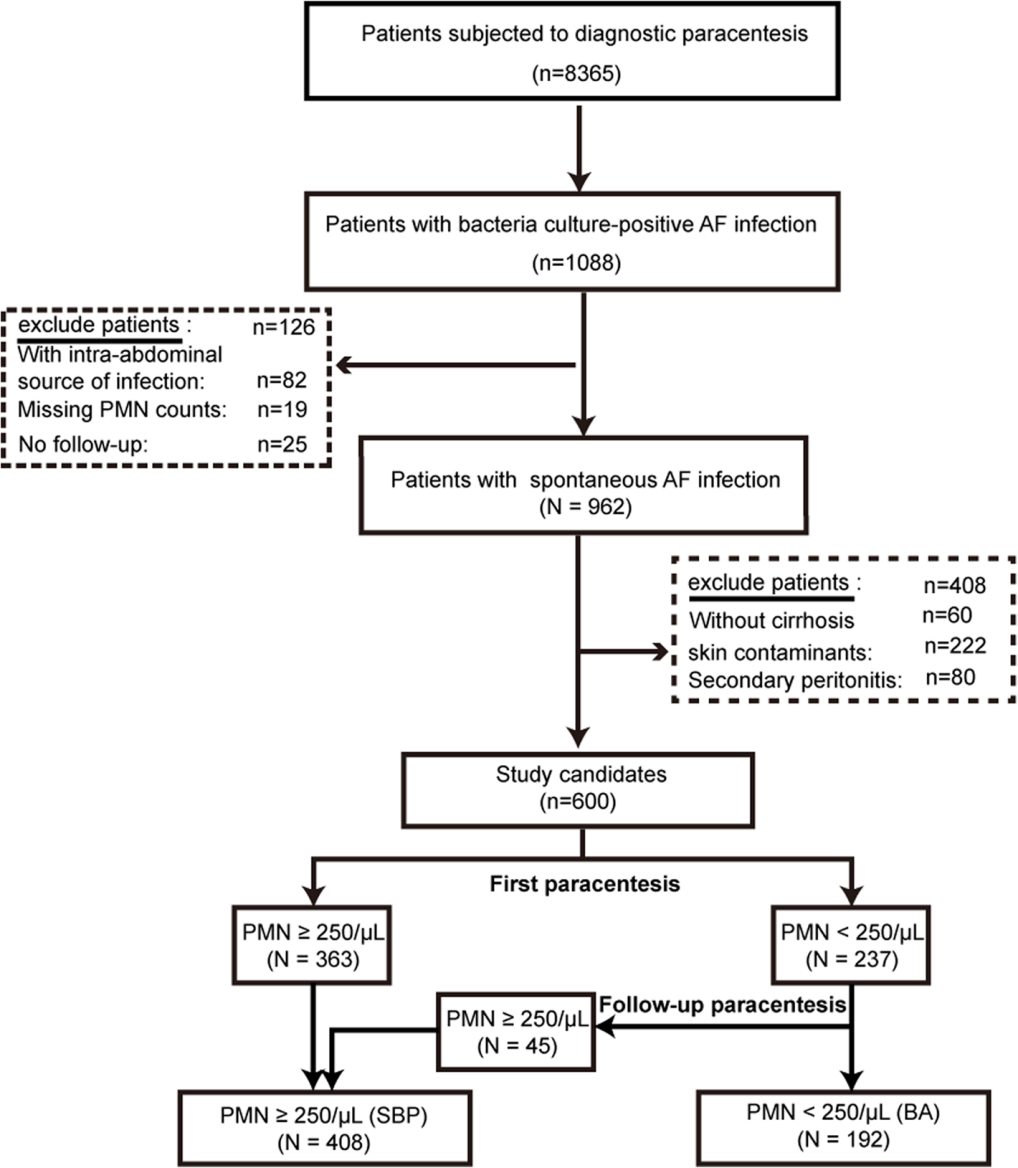
Flowchart of patient enrollment in the study. Abbreviation: AF, ascitic fluid; SBP, spontaneous bacterial peritonitis; BA, bacterascites; PMN, polymorphonuclear leucocytes

During the study period (January 1, 2012 to December 31, 2015), a total of 8365 patients were subjected to diagnostic paracentesis. Ascitic fluid culture in blood culture bottles was positive in 13.0% (*n* = 1088) of patients. The flowchart for patient enrollment was shown in Fig. 1, and only 600 ascitic fluid infection episodes (408 SBP and 192 BA) were identified in patients with cirrhosis and enrolled in this study (Fig. 1). Laboratory and clinical features at the time of diagnostic paracentesis were given in Table 1. Serum laboratory data (alanine transaminase/ALT, aspartate aminotransferase/AST, prealbumin, BUN, creatinine, total protenin, total bilirubin, erythrocytes/WBC, PMN and prothrombin time) and ascitic features (WBC and PMN) of SBP were significantly higher than that of BA (*p <* 0.01). In additional, septic shock was more common in patients with SBP (*p <* 0.01). There were no significant differences in the percentage of nosocomial episodes between SBP and BA.

**Table 1:** Clinical and laboratory data of cirrhotic patients with BA and SBP

	Total (*n* = 600)	BA (*n* = 192)	SBP (*n* = 408)	*p*
Sex (male)	465 (77.5%)	144 (75%)	321 (78.7%)	0.314
Age (yr)	53.9 ± 11.9	55.7 ± 12.1	53.1 ± 11.6	**0.011**
Presentation of symptoms	458 (76.3%)	118 (61.5%)	340 (83.3%)	**< 0.001**
Nosocomial infection	287 (47.8%)	84 (43.8%)	203 (49.8%)	0.17
MELD score	19.7 ± 9.5	16.3 ± 7.9	21.4 ± 9.8	**< 0.001**
Days in hospital	13 (7–19)	13 (7–18)	13 (7–19)	0.732
Days between admission and onset of infection	2 (0–9)	2 (0–6)	2 (0–10)	**0.03**
Causes of cirrhosis				
Hepatitis B	370 (61.7%)	105 (54.7%)	265 (65%)	**0.016**
Hepatitis C	59 (9.8%)	24 (12.5%)	35 (8.6%)	0.132
Biliary	22 (3.7%)	7 (3.6%)	15 (3.7%)	0.985
Alcoholic	90 (15%)	37 (19.3%)	53 (13%)	**0.044**
Others	59 (9.8%)	19 (9.9%)	40 (9.8%)	0.972
Serum features				
ALT(U/L)	35.0 (21.0–64.0)	30.0 (20.0–48.0)	38.0 (21.0–71.8)	**0.001**
AST(U/L)	60.0 (37.0–105.8)	53.0 (33.0–86.0)	65.5 (39.0–111.8)	**0.004**
Prealbumin(mg/L)	47.2 ± 25.8	55.3 ± 23.9	43.4 ± 25.8	**< 0.001**
Prothrombin time(S)	18.1 (15.0–22.6)	54.5 (40.2–68.0)	41.8 (26.0–57.7)	**< 0.001**
BUN(mmol/L)	8.0 (5.1–13.2)	6.4 (4.8–9.9)	8.9 (5.4–14.4)	**< 0.001**
Serum PMN(cells/μL)	5.4 (3.0–8.6)	4.0 (2.4–6.4)	6.2 (3.6–9.8)	**< 0.001**
Serum WBC(cells/μL)	6.9 (4.3–10.5)	5.4 (3.4–8.3)	7.7 (5.1–11.6)	**< 0.001**
Creatinine (μmol/L)	97.5 (78.0–138.8)	90.0 (70.2–111.8)	105.0 (82.0–157.0)	**< 0.001**
Total protein(g/L)	55.0 (49.0–61.0)	56.0 (51.0–63.0)	54.0 (48.0–60.0)	**0.001**
Total bilirubin (μmol/L)	82.4 (35.9–233.4)	51.5 (30.0–144.3)	107.9 (42.7–260.0)	**< 0.001**
Ascitic features				
Ascitic PMN(cells/μL)	799.5 (53.1–5405.8)	22.9 (5.6–71.3)	3099.9 (720.2–7873.0)	**< 0.001**
Ascitic WBC(cells/μL)	1241.0 (253.3–6565.0)	169.0 (90.3–310.8)	3850.5 (1119.3–9826.8)	**< 0.001**
Hepatocellular carcinoma	175 (29.2%)	49 (25.5%)	126 (30.9%)	0.178
Hepatic encephalopathy	203 (33.8%)	58 (30.2%)	145 (35.5%)	0.198
Septic shock	108 (18%)	19 (9.9%)	89 (21.8%)	**< 0.001**
Upper gastrointestinal bleeding	114 (19%)	34 (17.7%)	80 (19.6%)	0.580
Diabetes mellitus	101 (16.8%)	37 (19.3%)	64 (15.7%)	0.274
30-day mortality	131 (21.8%)	26 (13.5%)	105 (25.7%)	**0.001**

Abbreviation: *SBP* spontaneous bacterial peritonitis, *BA* bacterascites, *MELD* Model for End-Stage Liver Disease, *WBC* white blood cell, *PMN* polymorphonuclear leukocytes, *ALT* alanine transarninase, *AST* aspartate aminotransferase, *Bun* blood urea nitrogen

*P*:compared between BA and SBP. *P*-values < 0.05 are indicated in bold

### Bacteriological characteristics and impact of drug resistance on mortality

**Fig. 2:**
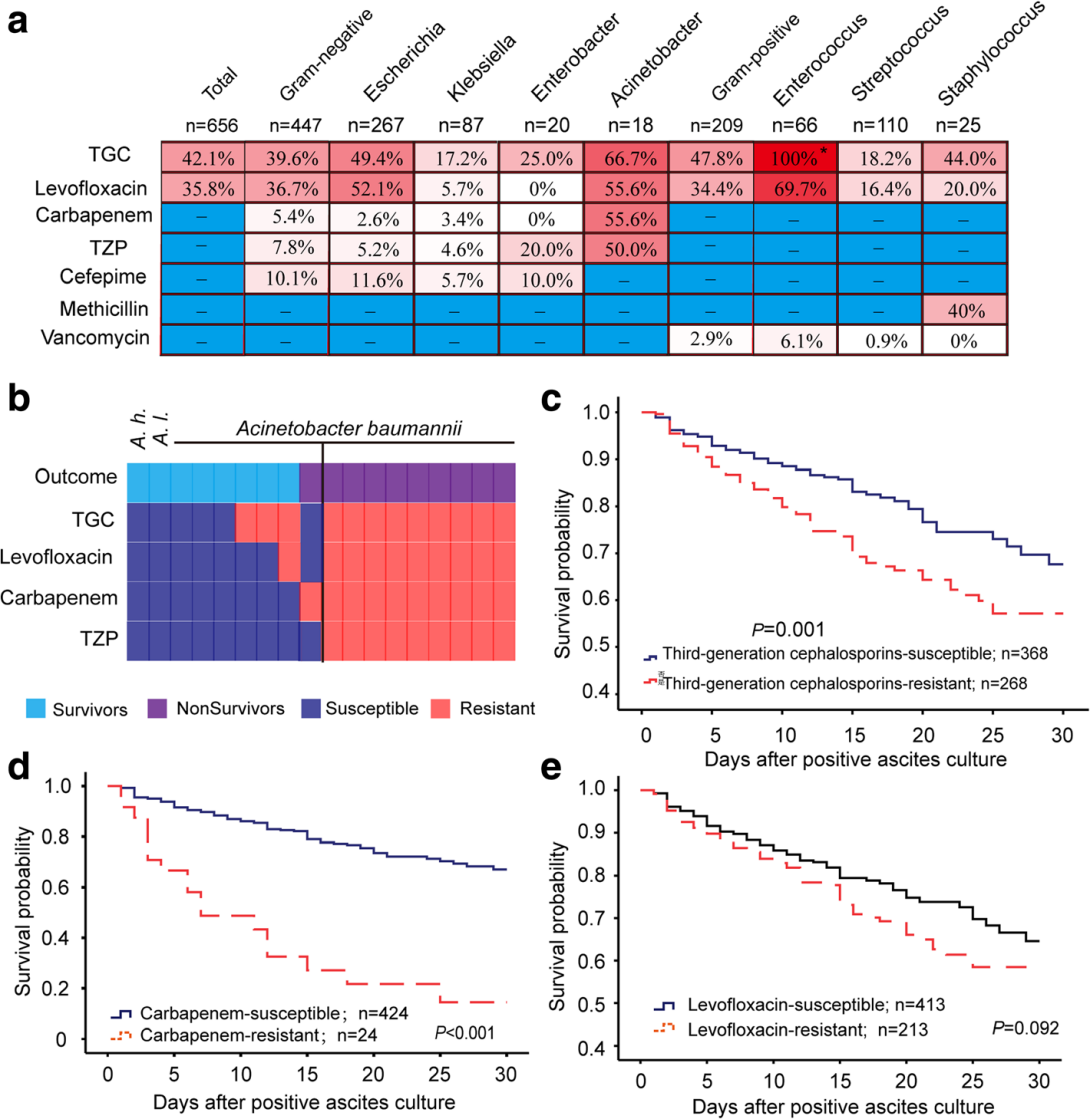
Drug resistance of major bacteria and impact of antibiotic resistance on mortality. (**a**) Resistance to antibiotics among seven major isolates. * *Enterococcus* spp*.* is naturally resistant to third-generation cephalosporins (TGC). TZP, piperacillin/tazobactam. (**b**) Effect of multi-drug resistance on patient mortality with respect to *Acinetobacter* spp. *A.h.*: *Acinetobacter haemolyticus*; *A.l.*: *Acinetobacter lwoffii.* (**c**, **d**, **e**) 30-day survival curve for patients with positive ascites culture according to third-generation cephalosporin, carbapenem or levofloxacin resistance (*p =* 0.004, < 0.001 and = 0.092, respectively)

Among the 600 patients, 554 patients had monobacterial infection and the other 46 had polybacterial infection. Thus, a total of 656 pathogens were isolated from these patients and shown in Table 2. Gram-negative bacteria, accounting for 68.1% of pathogens in total, were more common than gram-positive bacteria. The major pathogens identified were *Escherichia coli* (*n* = 267, 40.7%), *Streptococcus* spp. (*n* = 110, 16.8%), *Klebsiella* spp. (*n* = 87, 13.3%), *Enterococcus* spp. (*n* = 66, 10.1%), *Coagulase-positive staphylococci* (*n* = 25, 3.8%), *Enterobacter* spp. (*n* = 20, 3.0%) and *Acinetobacter* spp. (*n* = 18, 2.7%). In total, these 7 types of pathogens account for 90.4% (*n* = 593) of the causative bacteria. The antimicrobial susceptibility of these bacteria was shown in Fig. 2a. Nearly half of *Escherichia coli* (49.4%) were resistant to TGC, on the contrary, most of *Klebsiella* spp. isolates were sensitive to TGC. Vancomycin was a reliable agent for treating gram-positive pathogen infections because of the low resistant rates. Nine *Acinetobacter baumannii* strains were multidrug resistant. Multidrug resistant pathogens were associated with high 30-day mortality rate. Our data showed that patients infected with multidrug resistant *Acinetobacter* spp. had significantly higher 30-day mortality compared to patients infected with susceptible *Acinetobacter* spp. (100% vs. 11.1%, respectively, *p* < 0.001, Fig. 2b). Survival curves shows that patients infected with TGC-resistant bacteria had a significantly lower survival probability than those with TGC-susceptible bacteria (*p =* 0.001, Fig. 2c). Also, there was a significantly lower survival probability for patients infected with carbapenem–resistant organisms than it was in carbapenem-susceptible cases (*p <* 0.001, Fig. 2d). Levofloxacin-resistant organisms were not associated with greater mortality than levofloxacin-susceptible ones (*p =* 0.092, Fig. 2e).

**Table 2:** Types of bacteria isolated from cultures of ascitic fluid in patients with cirrhosis

Isolates	no.(%) of isolates			
	Total (*n* = 656)	BA (*n* = 208)	SBP (*n* = 448)	*P*
Gram-negative organisms				
Total	447 (68.1%)	116 (55.8%)	331 (73.9%)	**< 0.001**
*Escherichia coli*	267 (40.7%)	71 (34.1%)	196 (43.8%)	**0.020**
*Klebsiella* spp.	87 (13.3%)	20 (9.6%)	67 (15.0%)	0.061
*Enterobacter* spp.	20 (3.0%)	5 (2.4%)	15 (3.3%)	0.513
*Acinetobacter* spp.	18 (2.7%)	5 (2.4%)	13 (2.9%)	0.716
*Aeromonas* spp.	9 (1.4%)	2 (1.0%)	7 (1.6%)	0.726
*Citrobacter* spp.	8 (1.2%)	3 (1.4%)	5 (1.1%)	0.713
*Serratia* spp.	7 (1.1%)	2 (1.0%)	5 (1.1%)	1.000
*Burkholderia* spp.	5 (0.8%)	3 (1.4%)	2 (0.4%)	0.333
*Pseudomonas* spp.	8 (1.2%)	1 (0.5%)	7 (1.6%)	0.446
Other	18 (4.0%)	4 (1.9%)	14 (3.1%)	–
Gram-positive organisms				
Total	209 (31.9%)	92 (44.2%)	117 (26.1%)	**< 0.001**
*Streptococcus* spp.	110 (16.8%)	47 (22.6%)	63 (14.1%)	**0.006**
*Enterococcus* spp.	66 (10.1%)	29 (13.9%)	37 (8.3%)	**0.024**
*Coagulase-positive staphylococci*	25 (3.8%)	11 (5.3%)	14 (3.1%)	0.178
*Kocuria* spp.	3 (0.5%)	1 (0.5%)	2 (0.4%)	1.000
Other	5 (0.8%)	4 (1.9%)	1 (0.2%)	–

*P*:compared between isolates collected from BA and SBP. *P*-values < 0.05 are indicated in bold

### Risk factors for BA progressing to SBP

For 187 of 237 cases with ascitic fluid PMN counts < 250 cells/μL at the first paracentesis, a follow-up paracentesis was performed when culture results come back positive. In our hospital, if BA patient exhibited symptoms of peritonitis, the patient would be treated with empirical antibiotic immediately. The development of BA between the first and second paracentesis (usually 2–3 days) was of concern. Thus, potential risk factors for BA progressing to SBP were identified (Table 3). Results showed that development of BA was associated with the severity degree of liver diseases (MELD score, *p* < 0.001), serum PMN (*p* = 0.002), ascitic PMN (*p* = 0.002), presentation of fever (*p* = 0.036) and serious complications (hepatocellular carcinoma and septic shock, *p* = 0.035 and 0.014, respectively). In multivariate analysis, only the presentation of hepatocellular carcinoma (OR = 3.047, 95% CI:1.161–7.997, *p* = 0.024) and higher ascitic PMN count (OR = 1.007, 95% CI: 1.001–1.013, *p* = 0.023) remained independent predictors for BA progressing to SBP.

**Table 3:** Risk factors for BA episodes progressing to SBP

	Progress to SBP (*n* = 45)	No progress to SBP (*n* = 142)	Univariate analysis			Multivariate analysis	
			*P*	Odds ratio (95% CI)		*P*	Odds ratio (95% CI)
Sex (male)	37 (82.2%)	108 (76.1%)	0.390	0.687 (0.292–1.616)		–	–
Age (yr)	52.4 ± 9.2	55.4 ± 12.5	0.144	0.979(0.951–1.007)		–	–
Nosocomial infection	23 (51.1%)	57 (40.1%)	0.197	1.559 (0.795–3.059)		–	–
MELD score	23.0 ± 10.7	16.3 ± 7.4	**< 0.001**	**1.089 (1.046–1.133)**		0.503	1.036 (0.934–1.149)
Days between admission and onset of infection	3 (0–7.5)	1 (0–6)	0.239	1.018 (0.989–1.048)		–	–
Fever	30 (66.7%)	69 (48.6%)	**0.036**	**2.116 (1.049–4.268)**		0.053	2.533 (0.986–6.505)
Chills	4 (8.9%)	11 (7.7%)	0.806	1.162 (0.351–3.846)		–	–
Abdominal pain	15(33.3%)	34 (23.9%)	0.214	1.588(0.765–3.295)		–	–
ALT(U/L)	42.0 (24.0–106.0)	30.0 (20.0–49.0)	**0.011**	**1.005 (1.001–1.009)**		0.326	1.004 (0.996–1.011)
AST(U/L)	70.0 (42.0–135.5)	52.5 (33.0–88.5)	**0.030**	**1.003(1.000–1.005)**		0.602	1.001(0.996–1.006)
Prealbumin(mg/L)	41.3 ± 22.7	56.1 ± 24.8	**0.001**	**0.973 (0.957–0.989)**		0.224	0.986 (0.965–1.008)
Prothrombin time(S)	19.2 (15.7–31.1)	16.3 (14.1–19.3)	**< 0.001**	**1.078 (1.034–1.125)**		0.118	1.064 (0.984–1.149)
Bun(mmol/L)	8.6 (5.5–13.9)	6.6 (4.8–11.5)	0.074	1.039 (0.996–1.083)		–	–
Serum PMN(cells/μL)	4.9 (2.8–9.8)	4.2 (2.4–6.6)	**0.002**	**1.12 (1.042–1.205)**		0.161	1.641 (0.822–3.277)
Serum WBC(cells/μL)	6.1 (4.0–11.8)	5.4 (3.6–8.5)	**0.006**	**1.096 (1.027–1.17)**		0.223	0.671 (0.353–1.275)
Serum creatinine (μmol/L)	105.0 (82.0–159.0)	88.5 (70.8–113.5)	0.15	1.003 (0.999–1.006)		–	–
Total protein(g/L)	56.0 (50.0–61.0)	55.0 (50.0–63.0)	0.679	0.997 (0.983–1.011)		–	–
Total bilirubin (μmol/L)	98.5 (43.9–311.4)	51.9 (32.4–140.2)	**0.018**	**1.002 (1.000–1.005)**		0.36	0.998 (0.994–1.002)
Ascitic PMN(cells/μL)	64.7 (21.7–141.7)	24.0 (5.3–74.8)	**0.002**	**1.008 (1.003–1.013)**		**0.029**	**1.007 (1.001–1.013)**
Ascitic WBC(cells/μL)	257.0(114.5–370.0)	161.0 (91.8–302.8)	0.529	1.000(0.999–1.002)		–	–
hepatocellular carcinoma	20 (44.4%)	39 (27.5%)	**0.035**	**2.113 (1.056–4.229)**		**0.024**	**3.047 (1.161–7.997)**
hepatic encephalopathy	16(35.6%)	43(30.3%)	0.508	1.27(0.626–2.577)		–	–
septic shock	12(26.7%)	16 (11.3%)	**0.014**	**2.864 (1.235–6.639)**		0.799	0.851 (0.246–2.949)
Gastrointestinal haemorrhage	11 (24.4%)	29 (20.4%)	0.567	1.261 (0.57–2.786)		–	–
diabetes mellitus	7 (15.6%)	28(19.7%)	0.534	0.75 (0.303–1.856)		–	–

*P*-values < 0.05 and corresponding Odds ratio are indicated in bold

### Risk factors for 30-day mortality of patients with BA and SBP

The 30-day mortality of patients with SBP was significantly higher than that of BA (25.7% vs. 13.5%, *p* = 0.001) (Table 1). We studied independent risk factors of 30-day mortality in BA and SBP respectively, and found that independent risk factors were different between them. Multivariate analysis, including the variables associated with mortality by univariate analysis, showed that the concentration of BUN was independent risk factor for 30-day mortality of BA patients (Table 4). For patients with SBP, the independent risk factors for 30-day mortality were age, MELD score, septic shock and hepatocellular carcinoma (Table 5).

**Table 4:** Risk factors for 30-day mortality in patients with BA

	Survivors (*n* = 166)	Nonsurvivors (*n* = 26)	Univariate analysis			Multivariate analysis	
			HR (95% Cl)	*P*		HR (95% Cl)	*p*
Age (yr)	55.3 ± 12.2	58.3 ± 11.6	1.022(0.991–1.055)	0.162		–	–
Sex (male)	125 (75.3%)	19 (73.1%)	1.152 (0.484–2.742)	0.750		–	–
Nosocomial infection	68 (41%)	16 (61.5%)	2.21 (1.000–4.884)	0.050		–	–
MELD score	14.7 ± 6.8	26.1 ± 7.7	**1.153 (1.102–1.207)**	**< 0.001**		1.054 (0.988–1.125)	0.108
Days between admission and onset of infection	2(0–6)	4.5(0–17.3)	**1.022 (1.002–1.042)**	**0.027**		1.015 (0.986–1.045)	0.301
Symptoms of peritonitis	102 (61.4%)	16 (61.5%)	0.915 (0.414–2.022)	0.826		–	–
ALT(U/L)	28.0 (19.0–46.0)	45.5 (28.0–93.3)	**1.003(1.000–1.006)**	**0.036**		1.001(0.992–1.009)	0.861
AST(U/L)	50.0 (32.0–75.5)	88.0 (51.5–180.8)	**1.003(1.001–1.005)**	**0.002**		1.003(0.997–1.008)	0.308
Prealbumin(mg/L)	57.3 ± 24.2	42.6 ± 17.8	**0.975 (0.957–0.993)**	**0.006**		0.981 (0.958–1.004)	0.100
Bun(mmol/L)	5.9(4.7–9.1)	11.5 (7.7–15.5)	**1.099 (1.055–1.143)**	**< 0.001**		**1.1 (1.033–1.173)**	**0.003**
Serum PMN (cells/μL)	3.6 (2.1–5.8)	6.5 (5.2–12.6)	**1.219 (1.124–1.322)**	**< 0.001**		1.027 (0.926–1.14)	0.609
Total Protein(g/L)	57.0 (52.0–64.0)	51.0 (47.0–54.0)	0.979(0.957–1.001)	0.060		–	–
Ascitic PMN(cells/μL)	24.0 (6.5–69.0)	13.0 (3.2–115.0)	1.002 (0.996–1.008)	0.589		–	–
Hepatocellular carcinoma	42 (25.3%)	7 (26.9%)	1.056 (0.443–2.514)	0.903		–	–
Hepatic encephalopathy	39 (23.5%)	19 (73.1%)	**6.442 (2.704–15.346)**	**< 0.001**		2.382 (0.849–6.679)	0.099
Septic shock	8 (4.8%)	11 (42.3%)	**5.725 (2.614–12.536)**	**< 0.001**		2.247 (0.876–5.762)	0.092
Gastrointestinal haemorrhage	23 (13.9%)	11 (42.3%)	**2.966 (1.361–6.466)**	**0.006**		1.705 (0.655–4.438)	0.274
Diabetes mellitus	34 (20.5%)	3 (11.5%)	0.576 (0.1730–1.920)	0.369		–	–

*P*-values < 0.05 and corresponding HR are indicated in bold

**Table 5:** Risk factors for 30-day mortality in patients with SBP

	Survivors (*n* = 303)	Nonsurvivors (*n* = 105)	Univariate analysis			Multivariate analysis	
			HR (95% Cl)	*P*		HR (95% Cl)	*p*
Age(yr)	52.1 ± 11.5	55.9 ± 11.7	**1.025 (1.008–1.041)**	**0.003**		**1.021 (1.003–1.04)**	**0.022**
Sex (male)	245 (80.9%)	76 (72.4%)	1.326 (0.864–2.035)	0.196		–	–
Nosocomial infection	134 (44.2%)	69 (65.7%)	**2.311 (1.544–3.46)**	**< 0.001**		1.577 (0.946–2.627)	0.081
MELD score	18.7 ± 8.4	29.1 ± 9.5	**1.1 (1.078–1.122)**	**< 0.001**		**1.067 (1.04–1.095)**	**< 0.001**
Days between admission and onset of infection	2(0–7)	8(1–18.5)	**1.028 (1.016–1.039)**	**< 0.001**		1.015 (0.999–1.032)	0.07
Symptoms of peritonitis	259 (85.5%)	81 (77.1%)	0.672 (0.426–1.06)	0.088		–	–
ALT(U/L)	36 (21–63)	57 (25–150.5)	**1.001 (1–1.001)**	**0.002**		1.001 (1.000–1.002)	0.310
AST(U/L)	59 (37–100)	95 (48–202.5)	**1 (1–1.001)**	**< 0.001**		1.000 (1.000–1.001)	0.728
Prealbumin(mg/L)	46.8 ± 25.9	33.7 ± 22.9	**0.98 (0.971–0.989)**	**< 0.001**		0.999 (0.989–1.009)	0.791
Bun(mmol/L)	8 (5.1–11.9)	13.3 (8.1–20.8)	**1.061 (1.043–1.078)**	**< 0.001**		0.998(0.977–1.019)	0.824
Serum PMN (cells/μL)	5.8 (3.5–9.2)	7.5 (3.8–12.2)	**1.061 (1.032–1.091)**	**< 0.001**		1.001(0.97–1.034)	0.931
Total Protein(g/L)	55 (49–60)	51 (44–57.5)	**0.979(0.967–0.991)**	**0.001**		1.002 (0.988–1.016)	0.778
Ascitic PMN(cells/μL)	2760 (741.4–7821)	3717.2 (660.9–8356)	1 (1–1)	0.241		–	–
Hepatocellular carcinoma	83 (27.4%)	43 (41%)	**1.874 (1.268–2.771)**	**0.002**		**1.755 (1.14–2.703)**	**0.011**
Hepatic encephalopathy	80 (26.4%)	65 (61.9%)	**3.282 (2.213–4.866)**	**< 0.001**		1.533(0.972–2.42)	0.066
Septic shock	24 (7.9%)	65 (61.9%)	**7.624 (5.135–11.321)**	**< 0.001**		**4.169 (2.599–6.686)**	**< 0.001**
Gastrointestinal haemorrhage	47 (15.5%)	33 (31.4%)	**2.02 (1.337–3.05)**	**0.001**		1.243 (0.798–1.936)	0.337
Diabetes mellitus	48 (15.8%)	16 (15.2%)	1.044 (0.613–1.778)	0.875		–	–

*P*-values < 0.05 and corresponding HR are indicated in bold

### Impact of isolate type on clinical characteristics and mortality

To study the impact of isolate type on clinical characteristics and mortality, we limited the analysis to patients with monobacterial ascitic fluid infections. Clinical characteristics of patients infected with different types of bacteria were shown in (Additional file 1): Table S1. There were significant differences in clinical characteristics among the seven types of bacterial infection, for example the percentage of peritonitis symptoms, several clinical complications, type of peritonitis (SBP), nosocomial infection, serum and ascitic features, MELD score and days between admission and onset of infection (*p <* 0.01). To investigate the impact of isolate type on the 30-day mortality, Cox proportional hazard model was used to control confounding variables, such as MELD score, defining isolate type as dummy variable (Table 6 and Additional file 1: Table S2). In univariate analysis, patients infected with *Acinetobacter* spp., *Klebsiella* spp. and *Enterococcus* spp. had higher hazard ratios of 30-day mortality compared to those infected with *Escherichia coli.* Multivariate analysis showed that only patients infected with *Klebsiella* spp. had higher hazard ratio of 30-day mortality compared to those with *Escherichia coli* (Table 6)*.*

**Table 6:** Hazard ratios (HRs) for 30-day mortality of different types of bacteria against reference strains

Bacteria	Univariate analysis			Multivariate analysis	
	HR (95%Cl)	*P*		HR (95%Cl)	*p*
*Escherichia coli*	Ref			Ref	
*Coagulase-positive staphylococci*	0.298 (0.041–2.161)	0.231		0.399 (0.054–2.929)	0.367
*Acinetobacter* spp.	**3.881 (1.654–9.108)**	**0.002**		0.805 (0.306–2.118)	0.665
*Enterobacter* spp.	2.385 (0.944–6.025)	0.066		1.537 (0.572–4.131)	0.394
*Klebsiella* spp.	**2.092 (1.264–3.46)**	**0.004**		**1.888 (1.092–3.265)**	**0.024**
*Enterococcus* spp.	**2.842 (1.644–4.911)**	**< 0.001**		0.964 (0.513–1.813)	0.911
*Streptococcus* spp.	0.551 (0.259–1.171)	0.121		0.95 (0.437–2.066)	0.896

Note: HRs were analyzed by using proportional hazards Cox regression model. In multivariate analysis, HR was adjusted for clinical parameters. Only the major monobacteria were included in analysis. Detailed information was shown in Table S2. *P*-values < 0.05 and corresponding HR are indicated in bold

## Discussion

In this study, we retrospectively investigated the clinical and bacteriological characteristics of 600 SBP and BA patients, and studied the outcomes of these patients. To our knowledge, this is the most comprehensive and largest dataset of patients with ascitic fluid infection from China, and it permits statistical comparison and risk factor analysis for prognosis of SBP and BA.

Nearly half (47.8%) of the ascitic fluid infection were nosocomial-acquired. It could be explained in part by that patients enrolled in this study underwent frequent hospitalization before they were admitted to our department and experienced long hospital stays. Previous studies also reported high nosocomial infection rates in those patients [4, 19]. High rate of nosocomial-acquired SBP was not a common in China. Li *et al.* found that nearly two-thirds of SBP in cirrhotic patients was community-acquired in a hospital located in Zhejiang province of China [12].

In comparison with the data base of SBP, little data for BA is available. Previous researches indicated that many of patients with BA indeed were symptomatic and as a variant of spontaneous bacterial peritonitis [20, 21]. However, the difference between BA and SBP is uncertain and controversial because the lack of appropriate data sets and systematic analyses. In this retrospective study, we compared the clinical and bacteriological characteristics between SBP and BA. BA patients had lower 30-day mortality and less severe liver diseases than SBP patients, as evidenced by a lower serum ALT and AST and lower MELD scores, which is consistent with a previous report [21]. Symptoms of peritonitis, especially fever, and septic shock were less common in BA patients. In addition, gram-positive organisms, such as *Streptococcus* and *Enterococcus*, were more frequently found in BA than in SBP episodes. Our data also showed that hepatocellular carcinoma and higher ascitic PMN count were independent risk factors for BA episodes progressing to SBP. BA patients accompanied with hepatocellular carcinoma and higher ascitic PMN count thus should be optimal candidates for primary prophylaxis of SBP.

Consistent with previous studies, we also found that MELD score [22], the presentation of hepatocellular carcinoma [23] and presentation of septic shock [19] were independent risk factors for 30-day mortality at the time of SBP diagnosis. However, only the concentration of BUN were identified as independent predictive factor of 30-day mortality in patients with BA. To our knowledge, this is the first study to identify the independent risk factors of 30-day mortality in patients with BA. The independent risk factors of 30-day mortality were different between BA and SBP patients. The reason might be that SBP patients had higher MELD score and were thus sicker.

Prognosis of patients with ascitic fluid infection was influenced by bacterial antibiotic resistance. Our study suggested that resistant to TGC, which have been considered as first-line treatment of SBP [14, 24], were associated with lower survival probability. This result is consistent with previously studies [25–27]. Also, carbapenem-resistant gram-negative pathogen is a challenge for treating peritonitis. Our study showed that carbapenem-resistance is associated with significantly lower 30-day survival probability (*p* < 0.01). Thus, this can be a life-threatening factor for cirrhotic patients with ascitic fluid infection.

Gram-positive pathogens were increasingly recognized as important causative bacteria in patients with SBP and BA [6, 7, 27, 28]. However, the impacts of those causative bacteria on the outcome of patients with ascitic fluid infection are less well understood. Polymicrobial infection would be a confounding factor in studying the impact of isolate type on characteristic and outcome of ascitic fluid infection. For instance, previous study have shown that *Enterococci* were of low virulence and were often found as a secondary invader in polymicrobial infections and the clinical relevance of enterococcal peritonitis is subject of debate [29]. Thus, we limited our analysis to patients with monobacterial peritonitis when investigating the impact of isolate type on clinical characteristics and mortality. Reuken *et al.* also limited their analysis to monobacterial peritonitis and found that enterococcal SBP patients had a poorer prognosis than non-enterococcal SBP [6]. In our study, we only observed higher hazard ratio of 30-day mortality in patients infected with *Acinetobacter* spp., *Klebsiella* spp. and *Enterococcus* spp. compared to those infected with *Escherichia coli* in univariate analysis. After adjusting for clinical parameters, however, only *Klebsiella* spp. infection (HR = 1.888, 95%Cl, 1.092–3.265, *p* = 0.024) showed higher hazard ratio of 30-day mortality compared to that of *Escherichia coli* infection in multivariate analysis. Patients accompanied with *Klebsiella* spp. peritonitis seem to be associated with poorer prognosis even though those bacteria were less resistant against frequently used antimicrobial.

There were limitations in our study. First, our study was limited by being a single-center retrospective study. However, our hospital is a referral center for liver disease in the capital of China and many patients come from different regions of the country. Compared with a previous report [12], there were similar pathogen profiles and drug resistance in the two hospitals in China. The major pathogens in both hospitals were *E. coli*, *K. pneumoniae*, *Enterococcus* spp*.* and *S. aureus*. Furthermore, the resistant rate of gram-negative bacteria against TGC was both approximately 40%. Thus, to a lesser extent, our data may be representative in SBP patients in China. Second, patients with culture-negative SBP (ascitic PMN ≥ 250 cells/μl and a negative culture result) were not included in this study. Thus, our findings, such as independent risk factors of 30-day mortality in patients with SBP, could not be applied to those patients.

## Conclusions

In conclusion, there were significant differences between BA and SBP regarding clinical and bacterial characteristics and 30-day mortality. Presentation of hepatocellular carcinoma and higher ascitic PMN count were independent risk factors for BA progressing to SBP. The concentration of BUN were identified as independent predictive factors of 30-day mortality in patients with BA. For patients with SBP, the independent risk factors for 30-day mortality were age, MELD score, septic shock and hepatocellular carcinoma. The *Klebsiella* spp. related peritonitis is of concern, because those infections are associated with poorer outcome. Strict infection control must be implemented to control the spread of third-generation cephalosporin or carbapenem-resistant pathogens.

## Additional file

Additional file 1: **Tables S1.** Characteristics of patients with monobacterial ascitic fluid infection, by type of infecting bacteria. **Table S2.** Detailed information about hazard ratios (HRs) for 30-day mortality of different types of bacteria against reference strains. (DOC 90 kb)

## Abbreviations

ALT: Alanine transaminase;
AST: Aspartate aminotransferase;
BA: Bacterascites;
BUN: Blood urea nitrogen;
MELD: End-Stage Liver Disease;
PMN: Polymorphonuclear;
SBP: Spontaneous bacterial peritonitis;
TGC: Third-generation cephalosporins
WBC: White blood cell;

## References

[1] Arvaniti V, D’Amico G, Fede G, Manousou P, Tsochatzis E, Pleguezuelo M, Burroughs AK: Infections in patients with cirrhosis increase mortality four-fold and should be used in determining prognosis. Gastroenterology 2010, 139(4):1246–1256, 1256.e1241–1245. 10.1053/j.gastro.2010.06.019 20558165

[2] European Association for the Study of the L (2010). EASL clinical practice guidelines on the management of ascites, spontaneous bacterial peritonitis, and hepatorenal syndrome in cirrhosis. J Hepatol.

[3] Ricart E, Soriano G, Novella MT, Ortiz J, Sabat M, Kolle L, Sola-Vera J, Minana J, Dedeu JM, Gomez C (2000). Amoxicillin-clavulanic acid versus cefotaxime in the therapy of bacterial infections in cirrhotic patients. J Hepatol.

[4] Campillo B, Richardet JP, Kheo T, Dupeyron C (2002). Nosocomial spontaneous bacterial peritonitis and bacteremia in cirrhotic patients: impact of isolate type on prognosis and characteristics of infection. Clin Infect Dis.

[5] Angeloni S, Leboffe C, Parente A, Venditti M, Giordano A, Merli M, Riggio O (2008). Efficacy of current guidelines for the treatment of spontaneous bacterial peritonitis in the clinical practice. World J Gastroenterol.

[6] Reuken PA, Pletz MW, Baier M, Pfister W, Stallmach A, Bruns T (2012). Emergence of spontaneous bacterial peritonitis due to enterococci - risk factors and outcome in a 12-year retrospective study. Aliment Pharmacol Ther.

[7] Piroth L, Pechinot A, Di Martino V, Hansmann Y, Putot A, Patry I, Hadou T, Jaulhac B, Chirouze C, Rabaud C (2014). Evolving epidemiology and antimicrobial resistance in spontaneous bacterial peritonitis: a two-year observational study. BMC Infect Dis.

[8] Fernandez J, Acevedo J, Castro M, Garcia O, de Lope CR, Roca D, Pavesi M, Sola E, Moreira L, Silva A (2012). Prevalence and risk factors of infections by multiresistant bacteria in cirrhosis: a prospective study. Hepatology.

[9] Alexopoulou A, Papadopoulos N, Eliopoulos DG, Alexaki A, Tsiriga A, Toutouza M, Pectasides D (2013). Increasing frequency of gram-positive cocci and gram-negative multidrug-resistant bacteria in spontaneous bacterial peritonitis. Liver Int.

[10] Fiore M, Maraolo AE, Gentile I, Borgia G, Leone S, Sansone P, Passavanti MB, Aurilio C, Pace MC (2017). Nosocomial spontaneous bacterial peritonitis antibiotic treatment in the era of multi-drug resistance pathogens: A systematic review. World J Gastroenterol.

[11] Wang FS, Fan JG, Zhang Z, Gao B, Wang HY (2014). The global burden of liver disease: the major impact of China. Hepatology.

[12] Li YT, Yu CB, Huang JR, Qin ZJ, Li LJ (2015). Pathogen profile and drug resistance analysis of spontaneous peritonitis in cirrhotic patients. World J Gastroenterol.

[13] Gou YZ, Liu B, Pan L, Yu HT, Wang JP, Wang DC (2010). Pathogens of spontaneous bacterial peritonitis change in northern China. Saudi medical journal.

[14] Runyon BA (2009). Management of adult patients with ascites due to cirrhosis: an update. Hepatology.

[15] Rimola A, Garcia-Tsao G, Navasa M, Piddock LJ, Planas R, Bernard B, Inadomi JM (2000). Diagnosis, treatment and prophylaxis of spontaneous bacterial peritonitis: a consensus document. International Ascites Club. J Hepatol.

[16] Kamath PS, Wiesner RH, Malinchoc M, Kremers W, Therneau TM, Kosberg CL, D'Amico G, Dickson ER, Kim WR (2001). A model to predict survival in patients with end-stage liver disease. Hepatology.

[17] Merli M, Lucidi C, Giannelli V, Giusto M, Riggio O, Falcone M, Ridola L, Attili AF, Venditti M (2010). Cirrhotic patients are at risk for health care-associated bacterial infections. Clin Gastroenterol Hepatol.

[18] Wayne PA. Methods for dilution antimicrobial susceptibility tests for bacteria that grow aerobically; approved standard-ninth edition M07-A9. CLSI. 2012;

[19] Cheong HS, Kang CI, Lee JA, Moon SY, Joung MK, Chung DR, Koh KC, Lee NY, Song JH, Peck KR (2009). Clinical significance and outcome of nosocomial acquisition of spontaneous bacterial peritonitis in patients with liver cirrhosis. Clin Infect Dis.

[20] Chu CM, Chang KY, Liaw YF (1995). Prevalence and prognostic significance of bacterascites in cirrhosis with ascites. Dig Dis Sci.

[21] Runyon BA (1990). Monomicrobial nonneutrocytic bacterascites: a variant of spontaneous bacterial peritonitis. Hepatology.

[22] Tandon P, Kumar D, Seo YS, Chang HJ, Chaulk J, Carbonneau M, Qamar H, Keough A, Mansoor N, Ma M (2013). The 22/11 risk prediction model: a validated model for predicting 30-day mortality in patients with cirrhosis and spontaneous bacterial peritonitis. Am J Gastroenterol.

[23] Tsung PC, Ryu SH, Cha IH, Cho HW, Kim JN, Kim YS, Moon JS (2013). Predictive factors that influence the survival rates in liver cirrhosis patients with spontaneous bacterial peritonitis. Clin Mol Hepatol.

[24] EASL clinical practice guidelines on the management of ascites, spontaneous bacterial peritonitis, and hepatorenal syndrome in cirrhosis. Journal of hepatology 2010, 53(3):397–417. 10.1016/j.jhep.2010.05.004 20633946

[25] Hung TH, Tsai CC, Hsieh YH, Tsai CC, Tseng CW, Tsai JJ (2012). Effect of renal impairment on mortality of patients with cirrhosis and spontaneous bacterial peritonitis. Clin Gastroenterol Hepatol.

[26] Ariza X, Castellote J, Lora-Tamayo J, Girbau A, Salord S, Rota R, Ariza J, Xiol X (2012). Risk factors for resistance to ceftriaxone and its impact on mortality in community, healthcare and nosocomial spontaneous bacterial peritonitis. J Hepatol.

[27] Umgelter A, Reindl W, Miedaner M, Schmid RM, Huber W (2009). Failure of current antibiotic first-line regimens and mortality in hospitalized patients with spontaneous bacterial peritonitis. Infection.

[28] Bert F, Noussair L, Lambert-Zechovsky N, Valla D (2005). Viridans group streptococci: an underestimated cause of spontaneous bacterial peritonitis in cirrhotic patients with ascites. Eur J Gastroenterol Hepatol.

[29] Harbarth S, Uckay I (2004). Are there patients with peritonitis who require empiric therapy for enterococcus?. Eur J Clin Microbiol Infect Dis.

