# Supplementary material for: Clinical and bacteriological features and prognosis of ascitic fluid infection in Chinese patients with cirrhosis
Source: BMC Infect Dis. 2018 Jun 4;18:253. doi: 10.1186/s12879-018-3101-1 (PMC5987451; doi:10.1186/s12879-018-3101-1)
Supplement: Supplementary file 1 — Tables S1. Characteristics of patients with monobacterial ascitic fluid infection, by type of infecting bacteria. Table S2. Detailed information about hazard ratios (HRs) for 30-day mortality of different types of bacteria against reference strains. (DOC 90 kb) [file 12879_2018_3101_MOESM1_ESM.doc]

Additional File 1: Supplementary Tables

Tabe S1. Characteristics of patients with monobacterial ascitic fluid infection, by type of infecting bacteria.

| Characteristic | *Escherichia coli* | *Acinetobacter* spp. | *Enterobacter* spp. | *Klebsiella* spp. | *Enterococcus* spp. | *Streptococcus* spp. | *Staphylococcus aureus* | *P* |
| --- | --- | --- | --- | --- | --- | --- | --- | --- |
| no.(%) of isolates | 249(44.9%) | 11(2.0%) | 17(3.1%) | 74(13.4%) | 41(7.4%) | 98(17.7%) | 20(3.6%) | - |
| Age(yr) | 53±12.2 | 55.1±10 | 55.4±11 | 53.7±12.6 | 54.9±11.8 | 53.2±11.9 | 58.9±8.4 | 0.467 |
| sex(male) | 190(76.3%) | 10(90.9%) | 13(76.5%) | 58(78.4%) | 30(73.2%) | 74(75.5%) | 16(80%) | **0.032** |
| Nosocomial infection | 104(41.8%) | 9(81.8%) | 9(52.9%) | 32(43.2%) | 34(82.9%) | 42(42.9%) | 8(40%) | **0.001** |
| Days between admission and onset of infection | 2(0-8) | 16(3-43) | 3(0-14) | 1(0-9) | 6(4-15) | 2(0-5.3) | 0(0-7.3) | **0.001** |
| Ascitic PMN(cells/μL) | 2072(109-7287) | 56(25-1200) | 1264(127-13618) | 1370(200-5812) | 119(22-3941) | 157(21-1929) | 106(8-1119) | 0.362 |
| Serum PMN(cells/μL) | 5.1(3.0-7.7) | 8.7(5.1-24.4) | 4.8(2.0-12.5) | 6.3(3.9-9.3) | 6.9(3.9-13.9) | 4.7(2.7-8.1) | 4.2(1.7-7.3) | **0.009** |
| MELD score | 20.6±7.8 | 28.9±14.0 | 26.7±10.7 | 20.9±11.2 | 23.6±10.8 | 15.2±7.7 | 15.0±8.3 | **<0.001** |
| Total Protein(g/L) | 56.1±7.0 | 50.0±19.5 | 48.6±15.9 | 55.1±13.4 | 49.2±12.0 | 53.5±14.0 | 55.1±28.4 | 0.089 |
| Prealbumin(mg/L) | 43.1±22.3 | 39.6±23.4 | 53.9±35.9 | 43.3±22.4 | 44±31.4 | 55.4±22.5 | 61.0±34.7 | **<0.001** |
| Bun(mmol/L) | 9.2±5.6 | 22.6±15.2 | 16.8±13.3 | 11.6±10 | 15.9±10.7 | 9.5±7 | 11.5±9.8 | **<0.001** |
| ALT(U/L) | 39.0(22.0-71.3) | 83.0(32.0-163) | 48.0(23.5-91.5) | 33.0(21.8-67.5) | 22.0(14.5-58.5) | 28.0(18.0-47.0) | 32.0(22.3-51.8) | **0.006** |
| AST(U/L) | 68.5(41-112.8) | 108.0(32.0-217.0) | 102.0(34.5-210.5) | 56.5(33.0-87.0) | 57.0(37.0-167.0) | 44.0(30.0-83.0) | 50.0(34.0-86.3) | **0.001** |
| Symptoms of peritonitis | 210(84.3%) | 11(100%) | 14(82.4%) | 58(78.4%) | 30(73.2%) | 67(68.4%) | 12(60%) | **0.004** |
| Septic shock | 38(15.3%) | 5(45.5%) | 3(17.6%) | 16(21.6%) | 18(43.9%) | 5(5.1%) | 2(10%) | **0.001** |
| Hepatocellular carcinoma | 61(24.5%) | 4(36.4%) | 7(41.2%) | 26(35.1%) | 12(29.3%) | 27(27.6%) | 12(60%) | **0.023** |
| Hepatic encephalopathy | 82(32.9%) | 7(63.6%) | 7(41.2%) | 28(37.8%) | 23(56.1%) | 26(26.5%) | 5(25%) | **0.009** |
| Gastrointestinal haemorrhage | 49(19.7%) | 3(27.3%) | 3(17.6%) | 13(17.6%) | 11(26.8%) | 18(18.4%) | 5(26.3%) | 0.868 |
| 30-day mortality | 45(18.1%) | 6(54.5%) | 5(29.4%) | 23(31.1%) | 18(43.9%) | 8(8.2%) | 1(5%) | - |

Table S2. Detailed information about hazard ratios (HRs) for 30-day mortality of different types of bacteria against reference strains.
^A^ HRs were compared to patients infected with Escherichia coli. Only the major monobacterial were included in analysis.

| Characteristic | Survivors  (n=404) | Nonsurvivors  (n=106) | Univariate analysis | |  | Multivariate analysis | |
| --- | --- | --- | --- | --- | --- | --- | --- |
|  |  |  | HR (95%Cl) | *P* |  | HR (95%Cl) | *p* |
| Age(yr) | 53.0±11.8 | 56.2±12.1 | **1.02(1.004-1.036)** | **0.012** |  | **1.02(1.003-1.038)** | **0.025** |
| sex(male) | 313(77.5%) | 78(73.6%) | 1.133(0.736-1.745) | 0.571 |  | - | - |
| Nosocomial infection | 168(41.6%) | 70(66.0%) | **2.649(1.771-3.962)** | **<0.001** |  | **2.024(1.161-3.528)** | **0.013** |
| Days between admission and onset of infection | 2(0-6) | 7.5(0-18.3) | **1.026(1.016-1.036)** | **<0.001** |  | 1.007(0.991-1.022) | 0.406 |
| Ascitic PMN(cells/μL) | 718(47-4749) | 2071(138-7202) | 1(1-1) | 0.647 |  | - | - |
| Serum PMN(cells/μL) | 5.0(2.8-7.9) | 7.4(4.6-11.9) | **1.081(1.052-1.111)** | **<0.001** |  | 0.999(0.965-1.034) | 0.954 |
| MELD score | 17.7±8.0 | 29.1±8.8 | **1.118(1.095-1.14)** | **<0.001** |  | **1.08(1.053-1.108)** | **<0.001** |
| Total Protein(g/L) | 55.0(50.0-61.0) | 51.0(44.0-56.3) | **0.977(0.966-0.989)** | **<0.001** |  | 0.996(0.98-1.012) | 0.624 |
| Prealbumin(mg/L) | 49.7±24.4 | 34.5±23.2 | **0.975(0.966-0.984)** | **<0.001** |  | 0.999(0.988-1.01) | 0.871 |
| Bun(mmol/L) | 7.3(4.9-11.2) | 13.1(8.6-20.7) | **1.068(1.051-1.086)** | **<0.001** |  | 1(0.978-1.023) | 0.995 |
| ALT(U/L) | 33.0(21.0-53.5) | 54.0(21.8-132.0) | **1.001(1-1.001)** | **<0.001** |  | 1(0.999-1.002) | 0.35 |
| AST(U/L) | 56.0(34.5-90.5) | 93.5(50.8-218.0) | **1(1-1.001)** | **0.002** |  | 1(1-1.001) | 0.686 |
| Symptoms of peritonitis | 323(80.0%) | 79(74.5%) | 0.776(0.501-1.201) | 0.225 |  | - | - |
| Septic shock | 28(6.9%) | 59(55.7%) | 7.231(4.92-10.61) | **<0.001** |  | **3.351(2.096-5.357)** | **<0.001** |
| Hepatocellular carcinoma | 110(27.2%) | 39(36.8%) | **1.568(1.056-2.329)** | **0.026** |  | **1.884(1.215-2.921)** | **0.005** |
| Hepatic encephalopathy | 107(26.5%) | 71(67.0%) | **4.15(2.768-6.221)** | **<0.001** |  | **1.993(1.282-3.1)** | **0.003** |
| Gastrointestinal haemorrhage | 66(16.4%) | 36(34.0%) | **2.125(1.421-3.177)** | **<0.001** |  | 1.356(0.87-2.113) | 0.179 |
| Diabetes mellitus | 76(18.9%) | 16(15.2%) | 0.872(0.512-1.485) | 0.615 |  | - | - |
| Resistant to TGC | 152(37.6%) | 56(52.8%) | 1.262(0.969-1.643) | 0.084 |  | - | - |
| Resistant to Levofloxacin | 139(34.4%) | 51(48.1%) | 1.393(0.974-1.991) | 0.69 |  | - | - |
| Bacteria type^A^ |  |  |  |  |  |  |  |
| *Escherichia coli* | 204(81.9%) | 45(18.1%) | Ref |  |  | Ref |  |
| *Coagulase-positive staphylococci* | 19(95.0%) | 1(5.0%) | 0.298(0.041-2.161) | 0.231 |  | 0.399(0.054-2.929) | 0.367 |
| *Acinetobacter spp.* | 5(45.5%) | 6(54.5%) | **3.881(1.654-9.108)** | **0.002** |  | 0.805(0.306-2.118) | 0.665 |
| *Enterobacter spp.* | 12(70.0%) | 5(29.4%) | 2.385(0.944-6.025) | 0.066 |  | 1.537(0.572-4.131) | 0.394 |
| *Klebsiella spp.* | 51(68.9%) | 23(31.1%) | **2.092(1.264-3.46)** | **0.004** |  | **1.888(1.092-3.265)** | **0.024** |
| *Enterococcus spp.* | 23(56.1%) | 18(43.9%) | **2.842(1.644-4.911)** | **<0.001** |  | 0.964(0.513-1.813) | 0.911 |
| *Streptococcus spp.* | 89(91.8%) | 8(8.2%) | 0.551(0.259-1.171) | 0.121 |  | 0.95(0.437-2.066) | 0.896 |
